# CEREBROSPINAL FLUID MARKERS OF ALZHEIMER’S-RELATED PATHOLOGY RELATE TO AMCI AMONG PEOPLE WITH HIV

**DOI:** 10.1093/geroni/igad104.1684

**Published:** 2023-12-21

**Authors:** Judith Lobo, Erin Sunderman, Ben Gouaux, Scott Letendre, Laura Campbell, Mark Bondi, David Moore

**Affiliations:** University of California San Diego, San Diego, California, United States; San Diego State University, San Diego, California, United States; University of California San Diego, San Diego, California, United States; University of California San Diego, San Diego, California, United States; University of California San Diego, San Diego, California, United States; University of California San Diego, San Diego, California, United States; University of California at San Diego, San Diego, California, United States

## Abstract

Older people with HIV (PWH) are at-risk for Alzheimer’s disease (AD) and its precursor, amnestic mild cognitive impairment (aMCI). Identifying aMCI among PWH is challenging because memory impairment is also common in HIV-associated neurocognitive disorders (HAND). To investigate the utility of CSF AD pathology markers in differentiating aMCI from HAND, we assessed how these markers differentially relate to aMCI versus HAND status among PWH. Participants included 74 PWH (Mean age=48 [SD=8.5]; 87.4% male, 56.5% White) from the National NeuroAIDS Tissue Consortium who completed a neurocognitive test battery assessing seven cognitive domains and had CSF biomarker data for Aβ42, p-tau181, p-tau181/Aβ42 ratio, Neopterin and NfL. aMCI was defined as impairment (<1.0 SD below normative mean) on ≥2 memory outcomes among learning, delayed recall and recognition with at-least one recognition impairment required. HAND was defined as impairment (<1.0 SD below normative mean) in ≥2 cognitive domains. Separate linear regression models adjusting for demographic and HIV-related characteristics were used to examine how individual biomarkers relate to diagnostic status. Fifty-eight percent of participants were diagnosed with HAND, 50.5% were diagnosed with aMCI. CSF p-tau/Aβ42 ratio levels were higher (indicative of greater pathology) in PWH with versus without aMCI (β=.363, p=.001), but did not differ by HAND status. No other AD biomarker significantly differed by group status. Results indicate that the CSF p-tau181/Aβ42 ratio relates specifically to an aMCI-like profile among PWH, and, thus, may contribute to disentangling aMCI from HAND and informing the need for further diagnostic procedures.

